# Automated Video-Based Analysis Framework for Behavior Monitoring of Individual Animals in Zoos Using Deep Learning—A Study on Polar Bears

**DOI:** 10.3390/ani12060692

**Published:** 2022-03-10

**Authors:** Matthias Zuerl, Philip Stoll, Ingrid Brehm, René Raab, Dario Zanca, Samira Kabri, Johanna Happold, Heiko Nille, Katharina Prechtel, Sophie Wuensch, Marie Krause, Stefan Seegerer, Lorenzo von Fersen, Bjoern Eskofier

**Affiliations:** 1 Machine Learning and Data Analytics Lab, Department Artificial Intelligence in Biomedical Engineering, Friedrich-Alexander-Universität Erlangen-Nürnberg, 91052 Erlangen, Germany; philip.ps.stoll@fau.de (P.S.); rene.raab@fau.de (R.R.); dario.zanca@fau.de (D.Z.); samira.kabri@fau.de (S.K.); johanna.happold@fau.de (J.H.); heiko.nille@fau.de (H.N.); bjoern.eskofier@fau.de (B.E.); 2 Animal Physiology, Department Biology, Friedrich-Alexander-Universität Erlangen-Nürnberg, 91058 Erlangen, Germany; ingrid.brehm@fau.de (I.B.); katharina.prechtel@fau.de (K.P.); sophie.wuensch@fau.de (S.W.); marie.m.krause@fau.de (M.K.); 3 Computing Education Research Group, Department of Mathematics and Computer Science, Freie Universität Berlin, 14195 Berlin, Germany; stefan.seegerer@fu-berlin.de; 4 Nuremberg Zoo, 90480 Nuremberg, Germany; lorenzo@vonfersen.org

**Keywords:** animal welfare, animal behavior, deep learning, object detection, animal monitoring, behavior observation, *Ursus maritimus*

## Abstract

**Simple Summary:**

Every institution that keeps animals under human care must ensure animal welfare. To analyze the state of an animal, various measurements can be performed, such as blood analysis or fur condition scoring. They also need to be observed as often as possible to gain further insight into their behavior. Such observations are performed manually in most cases, which makes them very labor- and time-intensive and prevent them from being performed on a continual basis. We present a camera-based framework that provides automated observation of animals. The system detects individual animals and analyzes their locations, walking paths, and activity. We test the framework on the two polar bears of the Nuremberg Zoo.

**Abstract:**

The monitoring of animals under human care is a crucial tool for biologists and zookeepers to keep track of the animals’ physical and psychological health. Additionally, it enables the analysis of observed behavioral changes and helps to unravel underlying reasons. Enhancing our understanding of animals ensures and improves ex situ animal welfare as well as in situ conservation. However, traditional observation methods are time- and labor-intensive, as they require experts to observe the animals on-site during long and repeated sessions and manually score their behavior. Therefore, the development of automated observation systems would greatly benefit researchers and practitioners in this domain. We propose an automated framework for basic behavior monitoring of individual animals under human care. Raw video data are processed to continuously determine the position of the individuals within the enclosure. The trajectories describing their travel patterns are presented, along with fundamental analysis, through a graphical user interface (GUI). We evaluate the performance of the framework on captive polar bears (*Ursus maritimus*). We show that the framework can localize and identify individual polar bears with an F1 score of 86.4%. The localization accuracy of the framework is 19.9±7.6 cm, outperforming current manual observation methods. Furthermore, we provide a bounding-box-labeled dataset of the two polar bears housed in Nuremberg Zoo.

## 1. Introduction

Ensuring animal welfare is a key responsibility of any animal-keeping institution [1,2]. Animal welfare is defined to be the collective physical, mental and emotional state of an individual animal [2] and should be guaranteed 24 h a day, seven days a week, ideally from birth to death [3]. Examining animal welfare requires reliable, reproducible, and repeated assessment of welfare indicators [4]. In zoos, this is typically achieved by *observing the behavior* and by measuring *physiological* and *physical indicators*. Physiological indicators are, for example, adrenal hormones, glucocorticoid metabolites, or biochemical and hematological parameters. Physical parameters include coat or body condition scoring, gait parameters, or pedal and dental health [5,6,7,8,9].

Typically, behavioral observations in zoos are carried out using traditional methods through direct observation, either by keepers or biologists manually scoring behavior [9]. Depending on the observed species and the specific research question, different activities (e.g., walking, standing, lying, feeding, social as well as abnormal behaviors) are in the scope of the observation. However, of particular importance is to record the animal’s position in the enclosure over time. Analyzing the spatio-temporal changes in enclosure usage gives insight into an individual’s activity and inactivity patterns, proximity and distance towards conspecifics, and preferences in area usage. Therefore, observing an animal requires spotting it, identifying it, and locating its position on the enclosure map. Since manual pinpoint localization is not possible, enclosures are typically divided into suitable segments depending on the structure of the enclosure and the position of the observer [10]. The animals’ positions are manually assigned to the respective segment limiting the maximum accuracy of the location to the size of the chosen segments.

As manual observations are very time-consuming, they are usually only carried out for a few hours per day, severely limiting their conclusiveness [7,9]. This leads to a selective assessment of the behavior of the animal as the observation of a few hours does not allow a general assertion [9,11]. Human observers are prone to error, especially in long-duration observations, and may observe only a small group of animals or only individuals, depending on the method chosen. In addition, for some species, extensive training is needed to recognize individual animals, as many species lack distinct visual features. Additionally, the problem of subjectivity still exists since the behavioral measurement is highly dependent on the perceptual abilities of the observer and always leaves room for error [12]. It can be concluded that manually performed long-term studies of the behavior of individual animals are associated with extremely high effort and costs, and still do not enable continuous monitoring.

An alternative to traditional manual observation methods is the use of a video-based monitoring system, which overcomes the aforementioned limitations and allows insight into behavior on a 24/7 time scale. To be able to automate the whole manual observation process, such a monitoring framework must perform the same processing stages: (a) the animals must be detected in the raw video data and (b) the identity of each individual must be determined. In the third stage, depending on the method, (c) different information about the individual behavior can be assessed. In addition, for the present *zoo setting*, the framework needs to cope with some additional difficulties compared to a *laboratory setting*. It needs to be able to monitor animals in large enclosures with low camera resolutions and varying light conditions. As the positioning of the cameras needs to be adapted to the specific enclosure requirements, the viewing angle on the animals might vary. Therefore, detection and identification methods must be pose-invariant and robust to occlusions of parts of the animals. Additionally, the framework should be applicable to different species, hence using species-specific features such as unique coat patterns [13,14,15] for identification of individuals is not ideal.

Very few state-of-the-art approaches provide a solution for automating the whole manual observation process. Table 1 provides an overview of current video-based behavior monitoring frameworks. They are analyzed regarding the aforementioned specific challenges faced in the zoo setting. The frameworks that come closest to solving the problem under discussion are *Blyzer* [16], *idTracker* [17] and *GroupTracker* [18]. Blyzer is designed to detect animals of one species and outputs trajectories for further analysis. The image quality requirements for the camera are modest, yet the camera must be positioned to provide a top-down viewing angle. This specific positioning requirement of the camera as well as the lack of possibility to identify individuals severely limit the potential of this approach. The frameworks idTracker and GroupTracker are the only ones able to identify individuals for trajectory analysis. However, the limitation remains that these approaches only work in a laboratory setting. Only animals that remain visible in the same pose and show a high contrast to the background can be monitored. In summary, despite the great potential provided by recent deep learning developments, only a few frameworks exist that automate every step of the monitoring process, none of which is solving the specific challenges of the presented zoo setting. Our work aims to close this gap in research.

**Figure 1:**
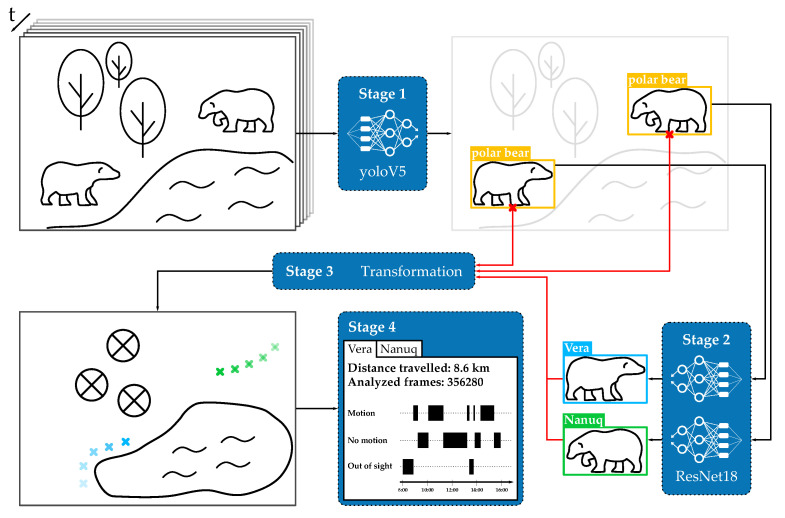
A high-level overview of the proposed framework. It takes raw videos as input and outputs labeled trajectories as well as basic statistics of the observed animal behavior. There are 4 major stages: **animal detection (1)**, **classification of individuals (2)**, **coordinate transformation (3)** from the image plane to the enclosure map and finally a **basic analysis (4)** of the trajectories.

To the best of our knowledge, we propose the first automated video-based framework for behavior monitoring of individual animals in a zoo setting. It is based on state-of-the-art deep learning algorithms and constitutes a step towards a non-invasive, fully automated animal observation system. Our framework takes raw videos of the animals in their enclosure as an input and outputs individual trajectories as well as basic statistics on the animals’ behavior. The framework consists of four main stages. First, the animals are located in the video (*object detection*, (1)) and the identity of each animal is determined (*classification*, (2)). Then, the positions of the animals are transformed from the camera plane to a map of the enclosure (*coordinate transformation*, (3)) for a meaningful interpretation. In the last step, the individual trajectories are analyzed (*trajectory analysis*, (4)). Finally, we present a graphical user interface that allows biologists and animal keepers easy access to the data and the statistics. A schematic overview of the proposed framework is depicted in Figure 1. Since no comparable framework exists [25], we compare the performance to previous manual observation methods.

We evaluate the proposed framework on polar bears (*Ursus maritimus*). This species is particularly challenging as individuals lack prominent distinct visual features. A limitation to this approach is that our study includes only two individual animals, which means that the classification problem is limited to two classes. However, polar bears are only kept with a few individuals in each zoo, making our approach representative of other institutions. Monitoring animal welfare of polar bears is of particular concern, as they are prone to abnormal behaviors under human care [6,26]. Skovlund et al. [27] analyzed 46 publications to identify and validate animal-welfare-based indicators for polar bears. Individual activity and inactivity patterns monitored over time and interpreted in context with husbandry and environmental conditions are identified as promising indicators for polar bears and are recommended for further research [27]. The framework we propose allows the first continuous monitoring of these parameters.

In summary, our contribution is a video-based framework explicitly designed to monitor individual animals in a zoo setting. For that, we use state-of-the-art deep learning models. We evaluate this framework on a newly created dataset of polar bears. Finally, we provide this extensively annotated dataset consisting of 4450 images, including a suitable method for aggregating annotations made by any number of experts.

## 2. Dataset

**Figure 2:**
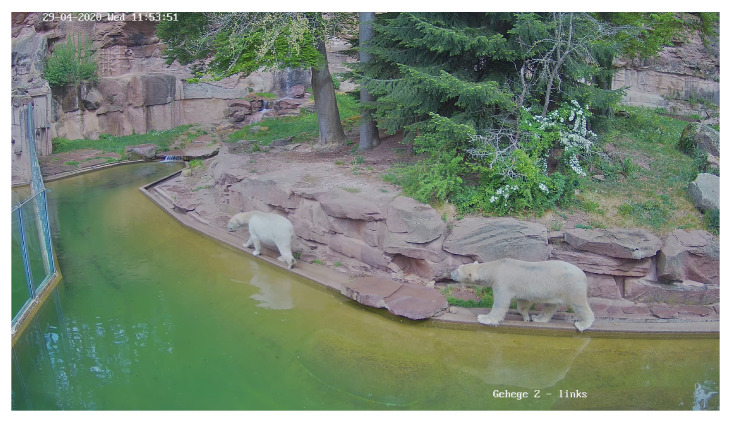
Example picture taken with one of the three cameras. Both animals are walking in one of the two outdoor enclosures. The polar bear on the left is Vera, the one on the right is Nanuq.

For the purposes of implementing and evaluating the proposed framework, a dataset consisting of 4450 images showing polar bears under human care was collected. Please note that while detection of polar bears could just exploit a pre-trained model, we still need to collect the data to perform the identification of individuals. The images have been taken at the polar bear enclosure at Nuremberg Zoo, which is home to two mature animals (*Vera*, female adult and *Nanuq*, male adult). An example image including both animals is shown in Figure 2.

### 2.1. Data Collection

The polar bear exhibit at Nuremberg Zoo consists of two indoor and two outdoor enclosures used to keep the polar bears seasonally separate (typically from August to February). However, the enclosures can be set up to allow the polar bears to share the outdoor areas during the mating season (March to June) or until intraspecific aggression occurs. Three video cameras continuously monitor both outdoor enclosures. They are aligned so that the visitor areas are not recorded, resulting in unrecorded areas where the animals’ behavior cannot be evaluated. The cameras acquire videos with a frame rate of 12.5fps and a resolution of 3840×2160 pixels. For the aim of this project, a period of five days of data (27 April–1 May 2020) has been selected. During this period, the polar bears shared both enclosures and thus might both be present in a single image. A total of 4450 frames were randomly selected and stored for further labeling. Three biologists annotated all images to provide labels of high quality by assigning labeled bounding boxes to the animals visible in the picture.

### 2.2. Accordance Metric for Multiple Annotators

Aggregating labels from multiple experts requires a suitable metric for annotation quality assessment. The most commonly used evaluation metric for bounding box annotations is the *Intersection over Union* (*IoU*) [28]. However, it can only be used to compare two annotated areas, e.g., a network prediction and a ground truth label. Literature provides some modified versions of the IoU metric for different purposes (e.g., [29] or [30]), none of which are applicable for our labeling setting with several competing biologists. Therefore, we propose a modified IoU-based accordance metric for competitive bounding box labeling of more than one expert with unique classes:

Consider K≥2 experts and M≥1 unique classes (e.g., animal identities). Every annotator k∈{1,…,K} creates a bounding box $B_{k,m}$ for each m∈{1,…,M}. If one class is not present in the image or the annotator does not find it, we consider an empty box. Pairwise comparison of two annotations of the same class *m* by two annotators *k* and *l* is provided using the IoU metric:

(1) $${\mathrm{IoU}}_{k,l,m}=\mathrm{IoU}\left(B_{k,m},B_{l,m}\right)=\frac{\left|B_{k,m}\cap B_{l,m}\right|}{\left|B_{k,m}\cup B_{l,m}\right|}$$

Based on this, we can calculate the accordance rate R∈[0,1] for each dataset instance. For each class m∈M, we calculate the respective pairwise IoU of two annotations and divide this by the number of all pairwise comparisons (*M* comparisons per class and (K−1)K/2 comparisons between the different observers) for normalization:

(2) $$R=\frac{2}{(K-1)K}\frac{1}{M}\sum_{k>l=1}^{K}\sum_{m=1}^{M}{\mathrm{IoU}}_{k,l,m}$$

R∈[0,1] is calculated for each instance, where R=1 is a perfect score meaning that all bounding boxes for each class align perfectly. In case of R=0 either the bounding boxes do not overlap, or the labels of overlapping boxes do not match.

Compared to the original IoU, the proposed accordance metric is suitable for situations in which multiple annotators compete for ground truth. Instances with *R* below a certain threshold (e.g., R≤1/|K|) should be discussed collaboratively.

### 2.3. Labeling Process

**Figure 3:**
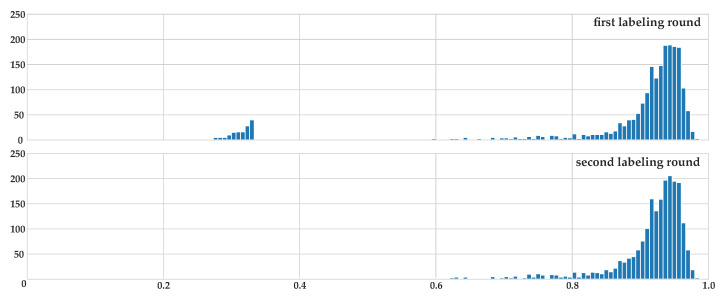
Accordance rate after first (**top**) and second (**bottom**) labeling round. The peak at R≈0.33 in the first labeling round is due to instances where only two of three experts found an animal, resulting in R≈0.33 when the pairwise agreement is computed. The same is true for the instances where all three experts found the same animal, but only two assigned the same identity. After the second collaborative round, this peak almost vanishes, implying a very high consistency in annotation for the dataset. Please note that instances without any animal (resulting in R=1) were excluded from this graph for a clearer presentation.

Annotation data collection was acquired in a two-step process performed by three trained biologists. In the first step, they labeled each image in a competitive process (using the EXACT labeling tool [31]), where each expert created bounding box annotations for each animal, including a label for their identities, not knowing about the annotations made by the other experts. The global accordance metric after the first labeling round was R=0.938. In the second stage, those instances with an accordance rate R<0.8 were collaboratively discussed. In the case of agreement, the labels were changed. After this process was finished, an overall accordance rate of R=0.958 was achieved, showing a very high consistency in the labeled dataset (see Figure 3).

### 2.4. Dataset Statistics

As the 4450 images were randomly selected from the video data, only 2099 instances show one or more animals. For most algorithms, empty images do not affect the training process, but may still be of value depending on the used method. Hence, the provided dataset also contains these images. 167 images show both animals, 1932 only one. 2266 bounding boxes are provided, 1082 for the male and 1184 for the female bear. We provide the data, including the label information under public license (see *Data Availability Statement*).

## 3. Framework

In this section, a detailed description of the proposed framework is provided. Figure 1 shows a high-level overview of the implemented framework. It takes raw videos as input and outputs labeled trajectories and basic statistics of the observed animal behavior. It consists of four major stages: object detection, classification of individuals, coordinate transformation from the image plane to the enclosure map and finally a basic statistical analysis of the trajectories.

### 3.1. Stage 1: Object Detection

Object detection is the task of estimating the location of a specific object in an image [32]. There are a variety of state-of-the-art algorithms for this task. The proposed framework uses the *yolo* algorithm [33] in the most recent implementation *yoloV5* [34], which performs a frame-based bounding box detection of the class *polar bears*. We chose this algorithm because it showed the best performance compared to other out-of-the-box object detectors in preliminary tests. The model is trained on the task of detecting polar bears using the bounding box annotations created by the three experts (without using the additional information about the identity of the respective animal).

### 3.2. Stage 2: Classification

After locating the polar bears within the images, the next step is to assign the individual identities. This allows determining valuable information about the behavior of each individual. Therefore, the task of the second stage of the framework is the classification of the animals with respect to their identities. For this purpose, the input images are cropped according to the detected bounding boxes (one polar bear per cropped image). The cropped images are then fed into the very common *ResNet18* deep neural network architecture [35], which has been trained on the identity annotations created by the three biologists.

### 3.3. Stage 3: Coordinate Mapping

**Figure 4:**
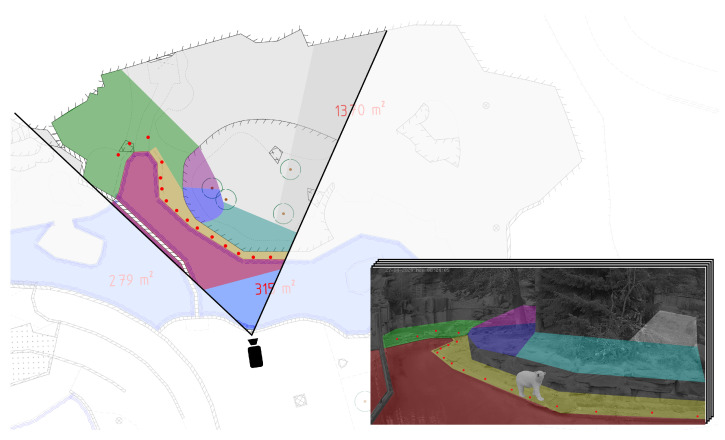
Schematic representation of the implemented coordinate transformation. The enclosure is divided into segments, which represent flat surfaces in good approximation. For each segment a homography matrix is determined, which then transforms the coordinates to the map of the enclosure.

The individuals can now be located on the camera plane for each frame. The center of the lower edge of the bounding box is defined as the animal’s position in the image. However, to allow for meaningful statements regarding enclosure usage and trajectories we need to assess the animal’s position on a map of the enclosure. Therefore, a coordinate transformation from the camera plane to the enclosure map plane must be performed. The underlying problem is a non-linear perspective transformation from the two-dimensional camera views of the three-dimensional terrain into a top-down view of the enclosure. In the first step, the camera planes are divided into 33 segments, each of which is a plane surface in reasonable approximation. This reduces the problem to a linear transformation for each segment, which can be performed by a homography transformation. To calculate the homography matrices for each segment, a sufficient number of points must be defined that can precisely be assigned both on the camera and the map layers. In total, 193 of these points were determined for the polar bear enclosures in the Nuremberg Zoo. An animal’s position on the enclosure map can be determined by first assigning the coordinates of an animal to a segment and then transforming the coordinates through a homography transformation. The whole process is depicted in Figure 4. By mapping the individual coordinates of the animals from a series of images to the enclosure plane, we receive their trajectories over time, which can be investigated using the graphical user interface for further analysis and graphical processing.

### 3.4. Stage 4: Analysis and Graphical User Interface

We provide a graphical user interface that allows viewing, interpreting, and exporting the data for further analysis. This user interface was developed in close collaboration with the biologists using the system and relying on manual observation to accommodate their specific needs. This led to the following main features implemented:

- **Localization representation**: the tool provides the location of the individual animals in form of *heat maps* or *trajectories*. These visualizations give insights into the frequency of a bear’s visit at a particular location, which may help identify sites of favor as well as changes in daily behavior.
- **Length estimation**: for the selected time frame, the tool provides the distance traveled by the individual animals. This allows for a comparison of behavioral stability and seasonality in the animals’ behavior and its reaction following management interventions, e.g., behavioral enrichment or separation of the individuals.
- **Motion/Resting time**: the tool aggregates motion and resting time for the individual animals. For biologists, this ratio in combination with other welfare metrics gives insight into the animals’ stress level.

## 4. Experiments

In the following, we describe the training and the evaluation of each individual component of the framework. We performed a total of six experiments. The first four experiments assess the performance of the deep learning components, which relate to the first two stages of the framework (detection and classification of the two animals). Experiments 5 and 6 investigate the quality of the coordinate mapping stage.

### 4.1. Object Detection and Classification

To evaluate the object detection and classification stages, we need to analyze the performance of the selected algorithms on the given annotated data. Only the 2099 non-empty images were used as empty images are not required during the training of the chosen algorithms. The performance in each of the four experiments on object detection and classification was measured across a day-wise five-fold cross-validation. This means that the network is trained with data acquired on four days and tested on images of the unseen fifth day. Table 2 shows the resulting data distribution. As we predict bounding boxes instead of only labels, the model’s output naturally does not match the ground truth pixel-by-pixel. We define a predicted bounding box as a valid detection if the IoU with respect to the ground truth exceeds a threshold ϵ. A suitable metric for assessing the performance in object detection and classification is the F1-score, i.e., the harmonic mean of precision and recall. This allows accounting for both relevant aspects of the performance. First, how many instances classified as *polar bears* are really *polar bears* and are therefore valid detections (precision). Second, how many of all *polar bears* in the dataset were correctly predicted (recall).

**Experiment 1** investigated the performance of the *yolo* algorithm as an object detector for the class *polar bear*. To this end, we evaluated the F1-score for each of the five folds with different threshold levels ϵ∈{0.25,0.5,0.75,0.95}. Each training ran for 70 epochs with a batch size of 64. Within one fold, the score was averaged on five individual runs of training, each of which with random initialization of the model parameters and data shuffling. The results are listed in Table 3.

**Experiment 2** assessed the performance of different state-of-the-art algorithms to classify the cropped polar bear images either as *Vera* or *Nanuq*. In addition, the inference time for each algorithm was evaluated. Each training ran for 20 epochs using early stopping with a patience of five epochs. Again, we conducted five runs of training with random data shuffling within each fold. Table 4 shows the results for each algorithm.

In **Experiment 3** the performance of the whole machine learning part of the proposed framework was investigated. For this we combined *yolo* as the object detection stage together with *ResNet18* as the classification stage. Furthermore, we analyzed the possibility of solely using *yolo* for both detection and classification of the two animals in **experiment 4**. The resulting precision, recall and F1 scores for both individual animals as well as their respective weighted average are listed in Table 5. The weighted averaged scores consider individual scores of each class with respect to the number of samples from that class.

### 4.2. Coordinate Mapping

In the third stage of the framework, the animals’ coordinates in the image plane are transformed to the enclosure map plane. In this process, two potential issues need to be considered:

- The predicted bounding box of the framework differs slightly in size and position with respect to the ground truth introducing an offset in the mapped coordinates.
- Coordinate transformation via homography matrices is limited in accuracy due to the positioning of the cameras in combination with the topography of the enclosure.

These aspects are addressed in experiments 5 and 6, respectively.

In **experiment 5** we investigated the first aspect by assessing the difference in distance between the predicted and the ground truth position. Therefore, we kept the procedure from experiment 1 but considered the deviation of the resulting positioning of the animal (center of the lower edge of the bounding box) instead of the bounding box in general. Next, both the animal’s predicted and ground truth coordinates were mapped to the enclosure using the homography matrices. Finally, the distance between these two points was measured. The average deviation was 19.9±7.6 cm. Please note that not including the classification stage of the framework is meaningful as it does not influence the positioning quality of the framework.

**Figure 5:**
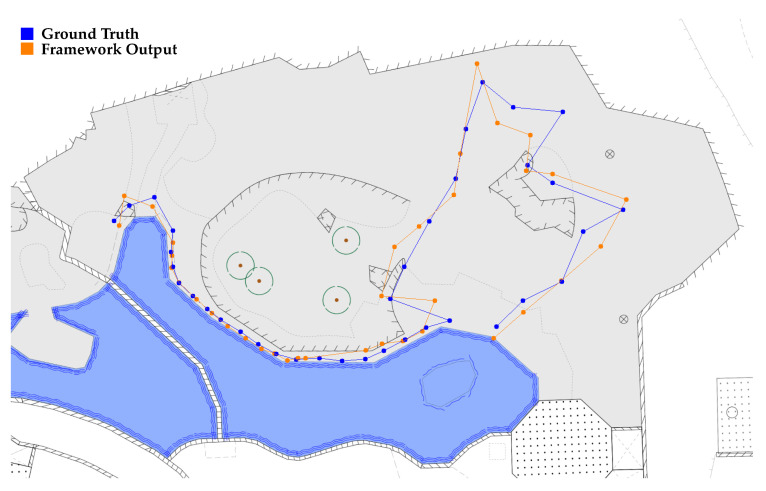
Graphical representation of the result of experiment 6. A person followed a trajectory while being tracked by the proposed framework. The output of the framework is shown in orange. At the same time, the person was positioned by two laser-based distance measuring devices. This trajectory, assumed to be ground truth, is depicted in blue.

In **experiment 6** we compared the quality of the implemented mapping algorithm with an alternative tracking method. A person followed a trajectory through both enclosures and was located at certain positions by two laser distance meters positioned outside the enclosure. At the same time, the person was filmed with the camera system and subsequently located with the proposed framework. The coordinates were transformed to the map plane and compared to the ground truth data given by the laser measurement. The trajectory for the larger enclosure is shown in Figure 5. In total, 104 positions in both enclosures were analyzed. The mean deviation from ground truth data is Δx=1.21±1.05 m. The ground truth trajectory in the left enclosure has a length of $S_{\mathrm{left},\;\mathrm{ground}\;\mathrm{truth}}=121.17$ m compared to $S_{\mathrm{left},\;\mathrm{measured}}=120.06$ m of the framework prediction. In the right enclosure, the laser measurement yielded a trajectory length of $S_{\mathrm{right},\;\mathrm{ground}\;\mathrm{truth}}=129.16$ m, whereas camera-based evaluation predicted $S_{\mathrm{right},\;\mathrm{measured}}=128.70$ m. Calibration tests showed that the laser measurement devices could achieve an accuracy of less than 10 cm in our experimental setting.

## 5. Discussion

The proposed framework’s performance was evaluated in six experiments. **Experiments 1** to **4** were designed to investigate the ability of the object detection and classification stages to find polar bears and identify individuals. **Experiments 5** and **6** assessed the quality of the coordinate mapping from the camera plane to the map of the enclosure. The results of all six experiments will be discussed in the following.

### 5.1. Object Detection and Classification

**Experiment 1** assessed the performance of *yolo* on the task of finding the class *polar bear* in the images. The F1 scores for the IoU thresholds 0.25, 0.50 and 0.75 are 0.942±0.022, 0.938±0.024 and 0.899±0.035, respectively – an acceptable performance regarding this project’s scope. The mean IoU over all folds is 0.784±0.044. As this results in a mean deviation of the position of 19.9±7.6 cm compared to ground truth (**experiment 5**), this IoU score is within a reasonable range for the aim of this project. As the mean IoU is 0.784, the F1 score for the IoU threshold of 0.95 is significantly lower at 0.227±0.075.

In **experiment 2** different state-of-the-art classification models were tested. The resulting F1 scores ranged from 0.832 (*ResNet101*) to 0.914 (both *ResNet18* and *MobileNetV2*). Since the presented framework can be used to evaluate large video data periods, the inference time needs to be considered. In this aspect, *ResNet18* showed the best performance, which is why this model was chosen for the final version of the framework. The models evaluated in this experiment showed the best performance in the first two folds in almost every run (see Table 4). A possible explanation for this is that the data shows no special features in the first two days. On the third day, the male animal stays comparatively often in areas very far away from the camera, while bushes often occlude the female. Both animals are recorded *standing* on this day. On day four, the male is again more often obscured by bushes. On day five, the image quality is negatively influenced by strong sunlight. Furthermore, both animals are swimming more often in the water area of the enclosure on this day. The described peculiarities explain the decrease of performance for the folds three to five because, on these days, incidents are shown, which the models did not see during training. Additionally, the fact that the performance decrease for these folds is within an acceptable range shows the ability of the framework and the individual models to generalize and deal well with unseen particularities.

**Experiments 3** and **4** analyzed the combined performance of the detection and classification stages. The precision for both experiments was >0.90 and thus very high. The framework consisting of *yolo* and *ResNet18* achieved a precision of 0.92. This means that for all instances predicted, only 8% are incorrect. These are the cases where either a polar bear is found but the wrong identity is assigned or an object from the background is incorrectly identified as a polar bear. In both cases, the outlier can be corrected by applying simple filtering methods since there is no spatio-temporal proximity to another detection of the same class. The influence of these erroneous instances on the overall performance of the framework is thus not problematic for the scope of this project. Please note that the precision increases for a higher IoU threshold. This is because the more precisely the animal is located during object detection, the better it can be classified.

The framework consisting of *yolo* and *ResNet18* achieves a recall of 83.2%. Thus, about 17% of all existing animal instances are not found. At a framerate of 12.5 frames per second, the information where the animals are located is missing only on 2–3 frames per second on average. This is not a problem and can be easily corrected by simple interpolation. The recall is about 5 to 6% better for experiment 3 compared to experiment 4. This means that using *yolo* alone results in about 5–6% fewer animals being found.

**Figure 6:**
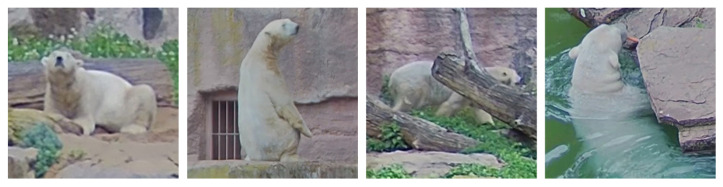
Difficult and unusual instances of the dataset. The first image shows Nanuq in a sandbox far away from the camera. The second image shows Nanuq *standing*. The third image shows Nanuq partly occluded. The last image shows Vera *swimming*.

The results show that by combining *yolo* (for object detection) and *ResNet18* (for classification) in experiment 3, as well as training *yolo* to solve both tasks simultaneously in experiment 4, we achieved F1 scores of more than 80%. However, *yolo* alone is about 3 to 4% less performant. It also shows that the male individual, Nanuq, is detected slightly less accurately. This is because the difficult instances for Nanuq occur more frequently than for Vera. He often lies in a sandpit that is distant from the camera. In addition, he is more often obscured or standing on his feet. Some examples of these difficulties are depicted in Figure 6.

Even though the difference in performance between the two approaches is rather small, using the two-step method still has its merits. One of the reasons is that the framework is designed for more accessible adaptation to new zoos. If another institution wants to use the framework, the labeling effort is reduced because the classifier is easier to train compared to the object detector, which can be used pre-trained as it is. Another argument for the two-stage approach is that *yolo* does not use the full resolution of the image due to its optimization for fast computation times. Classifiers such as *ResNet*, on the other hand, use the full resolution of the image. This approach is more reasonable for applying the framework in cases where more than two animals share an enclosure, and thus classification becomes more complex. It can be concluded that performance evaluation in experiments 1–4 shows that the first two stages of the framework can effectively detect and identify individual animals.

### 5.2. Coordinate Mapping

The main aim of the experiments on coordinate mapping was to assess the quality of localizing the animals within their enclosure. This stage aims to achieve the smallest possible deviation of the predicted position from the actual position, which can be defined as the center of the polar bear’s body. Due to the small number of cameras available and their limited viewing angles, the exact body center cannot be determined in every enclosure area. However, this deviation must always be considered in relation to the animal’s size. As male polar bears reach a length of 2.00–2.50 m from nose tip to tail tip [39], deviations within this order of magnitude do not significantly influence the quality of the coordinate mapping with regards to the overall objective of monitoring animal behavior. Additionally, we need to compare the frameworks positioning accuracy with previous manual observation methods. Since pinpoint localization is not possible with manual observation, enclosures are typically divided into either equidistant grids or suitable segments (depending on the specific conditions). The animals’ positions are then manually assigned to the respective area. When manually observing the polar bears at Nuremberg Zoo, the enclosure was thus divided into 34 segments with a mean width of 9.55±5.20 m.

Object of investigation in **experiment 5** was how the predicted bounding box of the framework deviates from ground truth. As we define the position of the animal to be the center of the lower edge of the bounding box, this deviation will also show in the transformed coordinates. The resulting mean deviation of 19.9±7.6 cm shows that this error is within the polar bears’ dimensions. It is also significantly more precise than previous applied manual observation methods, which achieve an average precision of 9.55±5.20 m when dividing the polar bear enclosures into segments. These results show that the deviation introduced by the object detection stage does not significantly affect the overall performance of the framework with respect to the biological research questions.

In **experiment 6** we investigated the systematical error induced by the coordinate mapping via the homography matrices. Compared to a reference measurement, obtained with two laser distance meters, the mean deviation of the points was Δx=1.21±1.05 m. Again, the deviation from the ground truth lies within the dimensions of the animal. Figure 5 shows that the error can be considered to be a constant offset in the respective segments of the enclosure and thus does not significantly contribute to the calculation of the total distance. When comparing the length of the ground truth trajectory to the length of the framework’s prediction, the difference was less than 1% for both enclosures. Thus, the deviation of the output of the framework from the actual position is almost neglectable for the calculation of the distance traveled. The error is only relevant when considering the probability distribution of the animal’s position in the enclosure. The induced offset depends on the enclosure area, as homography matrices are more precise in closer proximity and frontal plan view. Still, with an error of Δx=1.21±1.05 m, the enclosure usage can be analyzed more precisely compared to the mean precision of 9.55±5.20 m achieved with manual observation techniques.

A limitation introduced by this approach is that the topology of the enclosure is not incorporated into the trajectory calculation as there have not been enough cameras to provide any depth information.

In summary, the experiments show that no significant errors are introduced by this approach to coordinate mapping. The deviations are within a reasonable range with respect to the animal size. Furthermore, significantly more precise trajectories can be achieved than with previous manual observations. The possibility of determining the distances traveled by the animals is an insight into behavior that manual observation methods cannot provide. Thus, this method is suitable for effectively tracking the position of observed animals, even with a limited number of available cameras.

## 6. Conclusions

Measuring animal behavior is an important method in animal welfare research, especially when combined with physical and physiological parameters [7,9,27]. We propose a deep learning framework for non-invasive behavior monitoring of individual animals under human care. We provide a tool to indicate spatio-temporal usage of an individuals’ habitat area, which allows analysis of individual activity and inactivity patterns, and locomotion distances. These parameters are measured reliably, objectively, and repeatedly with a reproducible method. Therefore, the well-known limitations of animal behavior observation by human observers [7,9,25] concerning time restrictions and observer bias are overcome by our framework. Our experiments on polar bears show that the presented framework improves the current manual observation methods in all aspects. We allow biologists and animal caretakers to overcome time-consuming observation and thus to expand their datasets at a 24/7 time scale. This detailed insight into an animal’s daily routine is an important step towards ensuring animal welfare on a 24/7 time scale from birth to death [3].

Even if only basic behavior categories are analyzed, the data collected by the framework is of great use. For example, the effect of certain enclosure changes or management measures that aim to increase activity could be investigated with our continuous monitoring framework. Additionally, an analysis of individual activity and inactivity patterns throughout the year is an important part of analyzing behavior in relation to environmental influences such as temperature, day length, weather, or visitor numbers. Furthermore, physiological parameters such as the stress hormone cortisol, which can be measured retrospectively over weeks in hair [40], can be used in combination with behavioral data to better interpret the animal’s condition. Thus, this framework provides another essential part of the matrix available to analyze behavior precisely and objectively. As a next step, defining activity-inactivity-ratios or walking distances characteristic for a specific individual on a seasonal time base will make it useful as an early-warning system for animal keepers if unexpected changes in daily values appear. Thus, this framework also represents a suitable tool for evaluating welfare and enhances the interpretation of physiological data. Future work should investigate the transferability of this framework to a broad range of other individuals and animal species within different terrains. In particular, future studies might consider transferring our trained object detector model to other zoos to analyze the performance on other polar bears, which requires a re-training of the identification stage and thus labeling of a new data set. Although the framework itself is species- and enclosure-independent, the general performance will be influenced by the specific situation’s boundary conditions, including camera angle and resolution, species, and enclosure size. This should be an object of investigation.

## Figures and Tables

**Table 1:** Current video-based frameworks for animal behavior monitoring. They are listed according to the requirements that must be addressed for the present zoo setting: (a) species-unspecific approach; (b) identification of individuals; (c) applicable in the zoo setting (varying camera angles, low camera resolutions, varying light conditions, large enclosures); the last column lists the extracted behavioral features.

Framework	(a)	(b)	(c)	Output
	Unspecific	ID	Zoo	
ChickTrack [19]	✓	✗	✓	locomotion
Nakamura et al. [20]	✗	✗	✗	pose estimation
Swarup et al. [21]	✗	✗	✓	activity recognition
DeepLabCut [22]	✓	✗	✗	pose estimation
Nilsson et al. [23]	✓	✗	✗	count
Kashiha et al. [24]	✓	✗	✗	locomotion
Blyzer [16]	✓	✗	✗	trajectory
idTracker [17]	✓	✓	✗	trajectory
GroupTracker [18]	✓	✓	✗	trajectory
**Our Framework**	✓	✓	✓	trajectory

**Table 2:** Day-wise splitting of data. All images in the dataset were acquired in the same week from 27 April to 1 May in 2020. The second row states how many instances with polar bears were used (excluding empty images).

	Day					Total
	1	2	3	4	5	
**Images**	900	850	950	700	1050	**4450**
**Non-empty images**	477	419	406	383	414	**2099**

**Table 3:** Results of **experiment 1**. *yolo* was trained and evaluated in a five-fold cross-validation. The task is the detection of the class *polar bear*. The F1 score is calculated at different IoU thresholds ϵ∈{0.25,0.5,0.75,0.95} for the definition of a valid detection. Additionally, the mean IoU is given in the last row.

Metric	Fold					
	1	2	3	4	5	Overall
**F1 @0.25 IoU**	0.961	0.943	0.948	0.950	0.906	**0.942** ± **0.022**
**F1 @0.50 IoU**	0.961	0.940	0.944	0.947	0.897	**0.938** ± **0.024**
**F1 @0.75 IoU**	0.942	0.900	0.908	0.906	0.839	**0.899** ± **0.035**
**F1 @0.95 IoU**	0.194	0.225	0.232	0.338	0.146	**0.227** ± **0.075**
**Mean IoU**	0.824	0.786	0.794	0.807	0.709	**0.784** ± **0.044**

**Table 4:** Comparison of different state-of-the-art networks for image classification. The F1 score is given as a mean result of all runs of the day-wise five-fold cross-validation including the overall standard deviation. Inference time (IT) was evaluated on a single batch of size 8 on a *Nvidia GeForce RTX 2060*.

Architecture	F1 Score						IT [ms]
	1	2	3	4	5	Overall	
ResNet18 [35]	0.971	0.961	0.882	0.892	0.865	**0.914** ± **0.059**	3.7
ResNet50 [35]	0.956	0.944	0.890	0.846	0.796	**0.886** ± **0.076**	8.4
ResNet101 [35]	0.908	0.902	0.812	0.838	0.698	**0.832** ± **0.093**	15.5
MobileNetV2 [36]	0.972	0.963	0.894	0.850	0.888	**0.914** ± **0.052**	7.4
ResNeXt50 [37]	0.968	0.921	0.857	0.841	0.831	**0.884** ± **0.079**	12.3
DenseNet121 [38]	0.949	0.936	0.868	0.923	0.863	**0.908** ± **0.053**	20.0

**Table 5:** **Experiment 3** investigated the performance when using *yolo* for detecting the animals and *ResNet18* for classifying them. **Experiment 4** assessed the possibility of using *yolo* for both object detection and classification. For both experiments we evaluated precision, recall and resulting F1 scores at different thresholds for the IoU. The scores are given for both individual animals as well as the weighted average (w. a.).

Experiment	IoU Threshold	Precision	Recall	F1 Score		
				Vera	Nanuq	w. a.
**3**	0.50	0.920	0.832	0.900	0.842	**0.864**
	0.75	0.920	0.786	0.882	0.808	**0.844**
**4**	0.50	0.908	0.780	0.874	0.800	**0.836**
	0.75	0.910	0.728	0.856	0.748	**0.804**

## Data Availability

The data presented in this study are openly available as *Nuremberg Polar Bear Dataset* at https://doi.org/10.5281/zenodo.5910445.

## Abbreviations

The following abbreviations are used in this manuscript:

GUI	graphical user interface
IoU	Intersection over Union
yolo	you only look once (object detection framework)
